# Advanced Fluorometric Detection of Sulfathiazole Antibiotics in Food Samples with Molecularly Imprinted Polymer Coated CdTe Quantum Dots

**DOI:** 10.1021/acsomega.5c04765

**Published:** 2025-07-23

**Authors:** Bianca Mortari, Ademar Wong, Sabir Khan, Rosa Fireman Dutra, Maria Del Pilar Taboada Sotomayor

**Affiliations:** † Department of Analytical Chemistry, Institute of Chemistry, 28108 São Paulo State University (UNESP), Araraquara, São Paulo 14801-970, Brazil; ‡ National Institute for Alternative Technologies of Detection, Toxicological Evaluation and Removal of Micropollutants and Radioactives (INCT-DATREM), Araraquara, SP 14800-060, Brazil; § Technological Development Center—CDTec, Postgraduate Program in Materials Science and Engineering, PPGCEM/UFPEL, 28116 Federal University of Pelotas, UFPel, Pelotas, Rio Grande do Sul CEP: 96010-610, Brazil; ∥ Laboratory of Biomedical Engineering, Department of Biomedical Engineering, Federal University of Pernambuco, Recife 50679-901, Brazil

## Abstract

Sulfathiazole (STZ) is an antibiotic used for bacterial infections in humans and to boost farm animal health. Overuse can lead to harmful antibiotic residues in meat, posing risks to human health. This also contributes to the rise of antibiotic-resistant bacteria. Here we developed a fluorescent sensor for the detection and monitoring of sulfathiazole, utilizing molecularly imprinted polymers (MIPs) that possess selective cavities tailored to the target analyte. These MIPs were integrated with quantum dots—nanocrystalline semiconductors known for their fluorescent properties—resulting in a core@shell structure, referred to as QD@MIP. The synthesized materials were examined using a combination of advanced imaging and spectroscopic analysis method. Fluorescence analysis was used to optimize the acidity level and contact duration for QD@MIPs with STZ. With the conditions optimized, the sensor attained a linear detection range of 10 to 60 μg kg^–1^, establishing limit of detection value of 0.59 and 1.79 μg kg^–1^ for limit of quantification, respectively. The QD@MIP was tested for repeatability and reliability, showing relative standard deviation (RSD) values under 9%. Tests with four potential interfering substances confirmed the high specificity of the sensor, which also demonstrated effectiveness in real animal-derived food samples, achieving recovery rates above 80% for fortified STZ. This study demonstrates the potential of the QD@MIP sensor for accurate and reliable monitoring and analysis of food samples, showcasing its excellent performance and quality.

## Graphical abstract

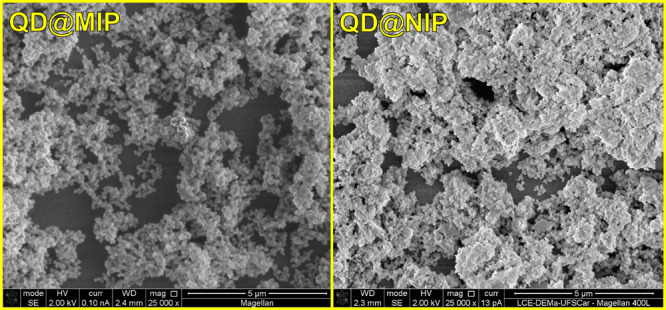

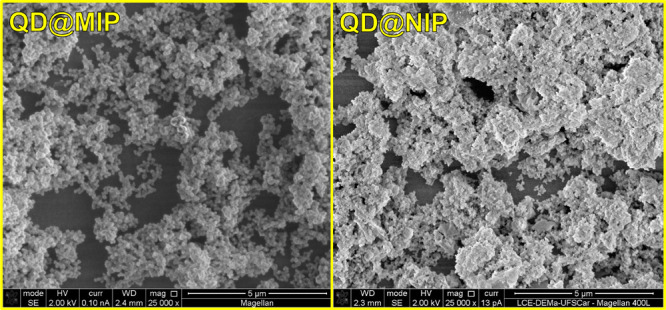

## 1 Introduction

Sulfonamides were the first drugs used to treat bacterial infections and were systematically applied for the selective treatment of specific bacterial diseases.^1,2^ Although these drugs were once widely used as common antibiotics, their use has drastically declined due to concerns about toxicity and the development of more effective alternatives. In some cases, sulfonamides are still employed for specific therapeutic purposes; however, their primary application has shifted to veterinary medicine.^3^ The drug sulfathiazole (STZ), discovered in 1939,^4^ was long used as a bactericidal agent, administered orally or topically to treat vaginal infections (e.g., gonorrhea) and skin infections.^5,6^ Although it has largely fallen out of use in human medicine due to the availability of less toxic and more effective drugs, it continues to be used in veterinary applications.^7^

The most distinctive characteristic of this compound is its polymorphism, which refers to its ability to crystallize into two or more distinct crystalline forms.^8^ Additionally, it exhibits tautomerism, in which its chemical structure exists in two different molecular forms that differ in the position of a proton (H^+^), as illustrated in Scheme 1C.^9^

**Scheme 1:**
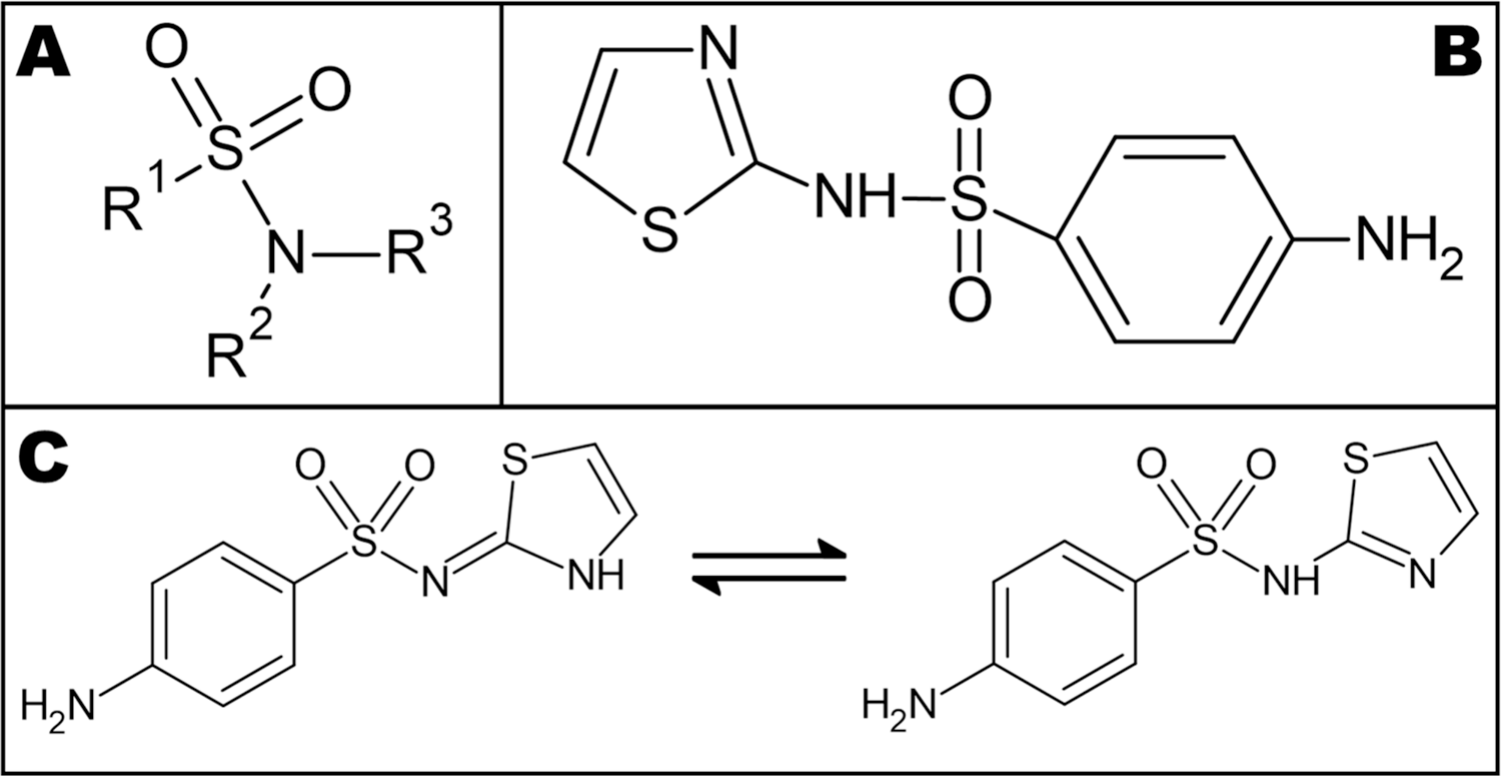
(A) Basic Structure Present in all Sulfonamides; (B) Sulfathiazole Structural Formula and (C) Sulfathiazole Tautomerism

The use of sulfathiazole in livestock has been prevalent for many years, serving to protect animals from various bacterial diseases and infections while also enhancing production. It is widely used in cattle to treat bacterial pneumonia, acute metritis, foot rot, and shipping fever complex.^10^

Compared to other antibiotics, sulfathiazole (STZ) does not pose significant risks to human health. However, its excessive use can lead to accumulation and generate unwanted residues in animal byproducts, as well as in feces and urine released into the soil, ultimately resulting in soil and groundwater contamination STZ possesses heterocyclic structures enriched with electrons and functional groups such as amino and sulfonamide, which can interact efficiently with fluorescent compounds. These interactions typically result in the suppression of fluorescence, mediated by mechanisms like photoinduced electron transfer (PET) or by static and dynamic quenching. Owing to this quenching effect, STZ can be detected with high sensitivity by observing the decline in fluorescence intensity of a selected fluorophore.^11^ The indiscriminate use of sulfathiazole or prolonged exposure to its residues may contribute to the development of antibiotic resistance in both veterinary and human contexts. Additionally, STZ is known for its bioaccumulative properties and relatively long half-life. Consequently, the widespread use of this antibiotic poses a risk of food contamination, and its metabolites may jeopardize consumer health.^12^ Some studies proved a great presence of accumulated STZ in animal products like meat, egg,^13^ milk,^14^ and honey.^15^ Although still used in some veterinary practices, does not have a Maximum Residue Limit (MRL) established by international regulatory agencies due to the lack of sufficient toxicological data to define safe levels. Therefore, developing analytical methods is very important for identifying and tracking the existence of sulfathiazole in animal food, to guarantee a better quality of their products and not compromise human and animal health.

In recent years, various studies have been conducted to develop efficient methodologies for the detection and quantification of target analytes. Molecularly Imprinted Polymers (MIPs) are an extraction and preconcentration technique based on highly selective synthetic materials. These polymers offer several advantageous characteristics, such as high selectivity, sensitivity, and low cost.^16,17^ MIPs exhibit a unique feature analogous to the “antigen–antibody” system, where the analyte acts as the “antigen” and the MIP functions as the “antibody.” This biomimetic approach reflects natural biological recognition mechanisms, hence MIPs are often classified as biomimetic polymers.^18^ The high selectivity and sensitivity of MIPs arise from the specific cavities formed during their synthesis, which possess shapes and sizes complementary to the target analyte.^19^ Moreover, MIPs can be fabricated in various shapes and sizes and can be combined with different materials to enhance their applicability and improve their properties, thereby broadening their use in the analysis of diverse analytes.^20^

Quantum dots (QDs) are nanoscale crystalline semiconductors with optical properties that depend on their size, shape, and composition, exhibiting high luminescence and photostability.^21^ One of the most notable features of QDs is their ability to emit bright, distinct colors when excited by an energy source (typically UV light), with the emitted wavelength varying according to the size and composition of the QDs, whose diameters range from one to several tens of nanometers.^22^ QDs can be combined with Molecularly Imprinted Polymers and are widely used as fluorescent probes. In this configuration, the polymer forms a layer around the semiconductor surface, creating a “core@shell” structure, where the quantum dot serves as the “core” and the MIP as the “shell” (QD@MIP).^23^ The QD@MIP material functions both as a fluorescent probe and a chemical sensor, particularly as a fluorescent optical sensor. The MIP shell provides a selective and sensitive surface capable of adsorbing specific analytes, while the quantum dot core interacts with these analytes through chemical or physical mechanisms. In recent years, QD@MIPs have garnered significant attention due to their advantageous properties, including the ability to selectively bind target molecules and facilitate efficient extraction and preconcentration directly at the QD@MIP interface.

In this work, a fluorescent probe was developed for the selective determination of the antibiotic sulfathiazole in milk, egg, and honey samples. The probe uses semiconductor quantum dots coated with molecularly imprinted polymers, combining the strong fluorescence of quantum dots with the specific recognition capabilities of MIPs. This nanocomposite enables efficient detection of sulfathiazole through fluorescence quenching, providing a promising tool for monitoring antibiotic residues in food matrices.

## 2 Experimental Section

### 2.1 Chemicals and Solutions

The materials used in the experiments, including tellurium (Te) powder (99.8%), were sourced from Sigma-Aldrich (SP, Brazil). Other reagents employed included sodium borohydride (NaBH_4_) (≥98.0%), 3-mercaptopropionic acid (MPA) (≥95%), cadmium chloride (CdCl_2_) (99.99%), sulfathiazole (STZ), tetraethyl orthosilicate (TEOS) (98%), (3-aminopropyl)­triethoxysilane (APTES) (99%), hydrochloric acid solution (37%), and sodium hydroxide pellets. Ammonia solution (28–30%) was provided by Emsure (BA, Brazil), while methanol and isopropanol were supplied by J.T. Baker (SP, Brazil). Additionally, tetracycline (≥98%), caffeine, chloramphenicol (≥98%), and hydrochlorothiazide were procured from Sigma-Aldrich. All solutions were prepared using deionized water obtained from a Millipore Milli-Q system, which ensures a resistivity of ≥ 18 MΩ·cm at 25 °C.

### 2.2 Apparatus

Fluorescence measurements were carried out using a Lumex Fluorat-02 Panorama spectrofluorometer, which was equipped with a xenon lamp and connected to a computer running version 2.3.4 of the Fluorat-02-Panorama (PanoramaPro) software. To characterize the molecular compositions of the materials and reagents, Fourier-transform infrared (FTIR) spectroscopy analyses were performed using a Bruker Vertex 70 spectrophotometer equipped with a DLaTGS detector, in the spectral range of 400–4000 cm^–1^. The physical characteristics of the polymer materials were analyzed using field emission gun scanning electron microscopy (FEG-SEM) with a JEOL model 7500 F, in conjunction with a confocal microscope from Tokyo, Japan.

Three magnetic stirrers were used for heating and mixing: Solad Model SL-91/A, Fisatom Model 751 (SP, Brazil), and Kasvi Model K45–1810H (PR, Brazil). Homogenization was accomplished with a Unique ultrasonic device (model USC-1850A, SP, Brazil) and a Norte Científica homogenizer (model NH 2200, SP, Brazil). Heating was carried out with a heating blanket provided by LGI Scientific (SP, Brazil). pH levels were measured using a Sensoglass pH meter (model SP1800, SP, Brazil). The separation of solids from the supernatant was conducted using a Kasvi Speedx1000 centrifuge (PR, Brazil).

Furthermore, fluorescence analysis was complemented by a confocal optical microscope (LEXT OLS 4000) with Olympus software to evaluate the surface texture and thickness of the materials.

### 2.3 Quantum Dot Synthesis

The quantum dot (QD) synthesis was performed in two parallel steps, following the methodology of Yang et al.^24^ with several modifications.

In the first step, 150 mL of ultrapure water was added to a round-bottom flask and purged with nitrogen gas for 10 min. Then, 216 mg of CdCl_2_ and 192 μL of MPA were added, forming a pale-colored solution. The pH was adjusted to 9 by adding 0.1 mol L^–1^ NaOH, resulting in a clear and transparent mixture. The flask was then placed on a heating mantle and maintained at 90 °C. Simultaneously, in a separate 25 mL Erlenmeyer flask containing 9.5 mL of ultrapure water, 40 mg of tellurium powder and 30 mg of NaBH_4_ were added. The solution was heated to 75 °C, stirred magnetically, and purged with nitrogen gas. After a few minutes, the solution turned purple and was immediately combined with the initial solution.

The resulting mixture turned reddish-orange and was stirred at 95 °C for 2 h. The CdTe quantum dots thus formed were purified by centrifugation with isopropanol in a 1:1 (v/v) ratio. The precipitate was redispersed in ultrapure water and stored at room temperature. The synthesis process is illustrated in Scheme 2A. The CdTe QDs were capped with MPA, whose thiol group binds to the quantum dot surface while the carboxylatec group (COO^–^) remains available for interaction with the polymer during MIP polymerization.

**Scheme 2:**
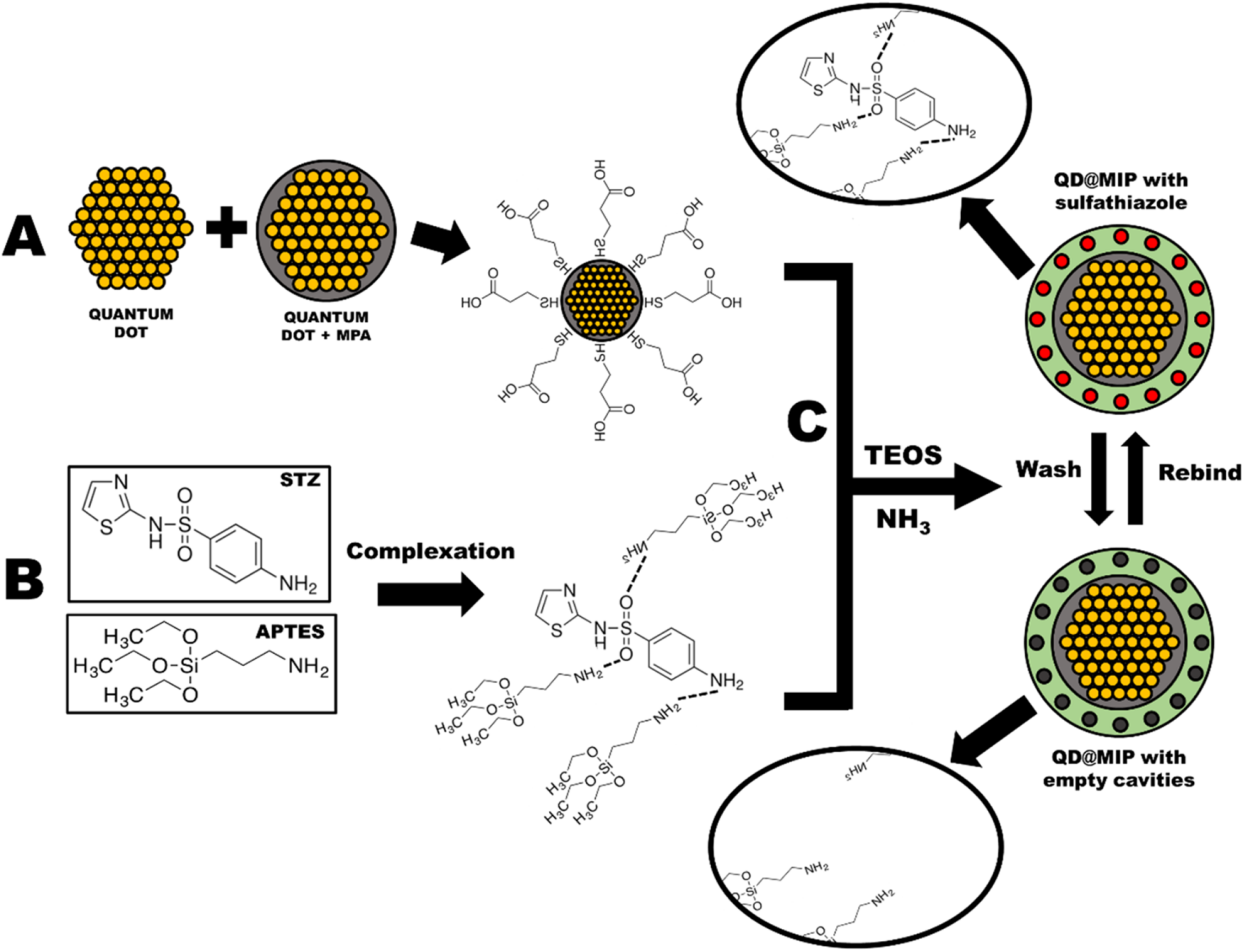
Schematic Illustration of the QD@MIP Synthetic Process for STZ Recognition^a^

a (A) Synthesis of MPA-coated QDs; (B) complexation between STZ (the template molecule) and APTES (the functional monomer); (C) final formation of QD@MIP, including STZ removal and subsequent rebinding to the molecularly imprinted cavities.

### 2.4 QD@MIP Synthesis

The development of the QD@MIP sensor was adapted from the research conducted by Yang et al.,^25^ with a few modifications. Initially, 0.6 mL of freshly prepared quantum dots (approximately 8 mg) was dissolved in 24 mL of ultrapure water. The solution was purged with nitrogen gas and magnetically stirred for 10 min.

Subsequently, the following reagents were added: 20 μL of APTES (functional monomer), 100 μL of TEOS (structural monomer), and 150 μL of ammonia (radical initiator), in a molar ratio of 1:5:7.5. The mixture was magnetically stirred and maintained in an oxygen-free environment at room temperature for 24 h. After this period, additional reagents were introduced: 20 μL of APTES, 5.7 mg of sulfathiazole, 50 μL of TEOS, and 30 μL of ammonia. The solution was stirred for another 24 h under the same conditions.

This process led to the formation of QD@MIP—a material consisting of CdTe quantum dots coated with a molecularly imprinted polymer. The resulting QD@MIPs were washed several times using a 1:1 (v/v) methanol–water mixture and separated by centrifugation. The purified particles were resuspended in 4.0 mL of ultrapure water and stored at room temperature, protected from light. A nonimprinted control material (QD@NIP) was also synthesized using the same procedure, except that sulfathiazole was omitted during the polymerization step.

The entire QD@MIP synthesis is illustrated in Scheme 2. Initially, CdTe quantum dots were synthesized with an MPA coating (Scheme 2A). This coating performs multiple roles: (i) providing anchoring sites for functional groups during MIP polymerization; (ii) minimizing cadmium ion leaching; (iii) protecting the QD core from external environmental factors; (iv) enhancing fluorescence stability; and (v) enabling hydrogen bonding interactions with STZ.

Scheme 2B shows how the template molecule (STZ) and APTES (functional monomer) form a noncovalent strong complex, which is then mixed with the quantum dots and polymerization reagents. In Scheme 2C, the TEOS (structural monomer) and NH3 (cross-linker) are added to synthesize the QD@MIP. After polymerization, the final product is washed to create cavities that match the shape and size of the template molecule, sulfathiazole. The interactions between sulfathiazole and the molecularly imprinted polymer using APTES occur mainly through hydrogen bonding between the amino groups of APTES and the sulfonamide groups and other polar regions of STZ, along with polar interactions and van der Waals forces that contribute to the MIP’s selectivity and affinity for the drug.

Nonimprinted polymers (NIPs) were synthesized in a manner analogous to molecularly imprinted polymers (MIPs), but in the absence of the template molecule during the polymerization process. As a result, NIPs do not contain specific recognition cavities within their polymer matrix. Their primary application lies in serving as control materials in comparative studies, enabling the evaluation of the selectivity and adsorption efficiency of MIPs, as well as distinguishing between specific and nonspecific interactions involved in molecular recognition processes.

### 2.5 Optimization Parameters of the Measurements

It is essential to obtain preliminary data from the synthesized materials prior to the main analyses. Initially, the pristine quantum dot was analyzed using a fluorimeter to determine its excitation and emission wavelengths, which were found to be 300 and 525 nm, respectively.

The measurement procedure of the study followed these steps: First, a 60 μg kg^–1^ stock solution of STZ was prepared in distilled water. From this stock solution, six aliquots of 3.0 mL each were taken to obtain final concentrations of 10, 20, 30, 40, 50, and 60 μg kg^–1^. Each aliquot was transferred into a sealed flask, to which 100 μg of QD@MIP was added. The flasks were tightly closed and placed in a homogenizer to promote interaction between STZ and QD@MIP. The pH and interaction time (rebinding time) were optimized for this step. After the STZ-QD@MIP (or QD@NIP) interaction, each solution was transferred to a cuvette for analysis, where excitation and emission spectra were measured using a fluorometer.

### 2.6 Evaluation of Duration of Interaction and pH Impact

To determine the optimal interaction time between the sulfathiazole (STZ) solution and the QD@MIP sensor, incubation periods of 5, 10, 15, 30, 60, 90, and 120 min were evaluated. For each time interval, 3.0 mL aliquots of a 20 μg·kg^–1^ STZ solution were prepared, and measurements were performed in triplicate following the established protocol.

In addition to incubation time, the effect of solution pH on the interaction efficiency between STZ and QD@MIP was investigated. Solutions were adjusted to pH values of 3, 5, 7, 9, and 11 using 0.1 mol·L^–1^ hydrochloric acid for acidification and 0.1 mol·L^–1^ sodium hydroxide for basification. All measurements were conducted at room temperature.

### 2.7 Development of the Analytical Curve

Following optimization of the rebinding time and solution pH, the QD@MIP sensor was evaluated to construct an analytical calibration curve, determine the corresponding equation, and calculate the limits of detection (LOD) and quantification (LOQ). Six STZ standard solutions at concentrations of 10, 20, 30, 40, 50, and 60 μg·kg^–1^ were prepared in triplicate using water as the solvent, and measurements were conducted according to the previously described protocol.

### 2.8 Evaluation of QD@MIP Specificity

Selectivity was evaluated using four potential interfering substances: tetracycline and chloramphenicol (antibiotics), hydrochlorothiazide (a diuretic), and caffeine. These compounds were selected due to their similar fluorescence quenching properties to sulfathiazole. Solutions of each interferent were prepared at concentrations of 10, 30, and 60 μg·kg^–1^ in the optimized pH medium, and all analyses were performed in triplicate.

### 2.9 Assessment of the QD@MIP in Food Product Samples

After characterization, parameter optimization, and construction of the analytical calibration curve, the rebinding performance of the QD@MIP sensor was evaluated using animal-derived food samples (milk, eggs, and honey) purchased from a supermarket in Araraquara, São Paulo State, Brazil. Each sample was subjected to a specific pretreatment procedure, described in detail below.

The milk sample pretreatment was adapted from Pizan-Aquino et al.^25^ with some modifications. To improve analytical performance, fat content was minimized by using skimmed milk. The milk was diluted 1:100 (v/v) with distilled water and centrifuged approximately four times to further reduce residual fat.

The honey sample was treated following the method described by Wang et al.^26^ Briefly, 1.0 g of honey was dissolved in 100 mL of water, heated in a water bath at 50 °C for 10 min, and subsequently centrifuged at 4000 rpm for 15 min. The resulting supernatant was filtered through qualitative filter paper.

The subsequent phase involves analyzing the repeatability and reproducibility of the proposed QD@MIP. Repeatability was assessed to confirm that the results from multiple consecutive measurements with the same sensor were consistent under optimized conditions.^29^ All analyses were conducted over ten consecutive measurements for each concentration of both polymers within a brief time frame. The relative standard deviation (RSD) was calculated using the following equation

$$\mathrm{RSD}=\frac{s}{\mathrm{x̅}}100$$

Were the “*s*” are the standard deviation and “*x̅*” are the mean of fluorescence variation values. The results are presented in Table S1, while the data pertaining to the fluorescence variation curve in relation to concentration can be found in Figure S2, both of which are included in the Supporting Information.

Egg sample pretreatment was based on Wen et al.,^27^ with modifications. Egg yolk and white were combined and homogenized. Then, 500 mg of the mixture was diluted in 10 mL of water and subjected to sonication for 10 min. The sample was centrifuged at 4000 rpm for 5 min, and the supernatant was filtered through filter paper. This centrifugation and filtration cycle was repeated four additional times to ensure thorough purification.

After completing the pretreatment procedures, each sample was transferred to a 50.0 mL volumetric flask and spiked with sulfathiazole to achieve a final concentration of 60 μg·kg^–1^, thereby preparing matrix-matched solutions for each food sample. Subsequently, aliquots of 3.0 mL from these solutions were placed into capped plastic flasks, with STZ concentrations adjusted to 10, 30, and 60 μg·kg^–1^ for each matrix.

## 3 Results and Discussion

### 3.1 Morphological and FTIR Analysis of Polymers

Field emission scanning electron microscopy (FEG-SEM) was employed for the morphological characterization of the materials, complemented by conventional SEM imaging. Analysis of the micrographs, presented in Figure 1, revealed uniformly distributed agglomerates with an average particle diameter of approximately 300 nm, as determined using SEM analysis software.

**Figure 1:**
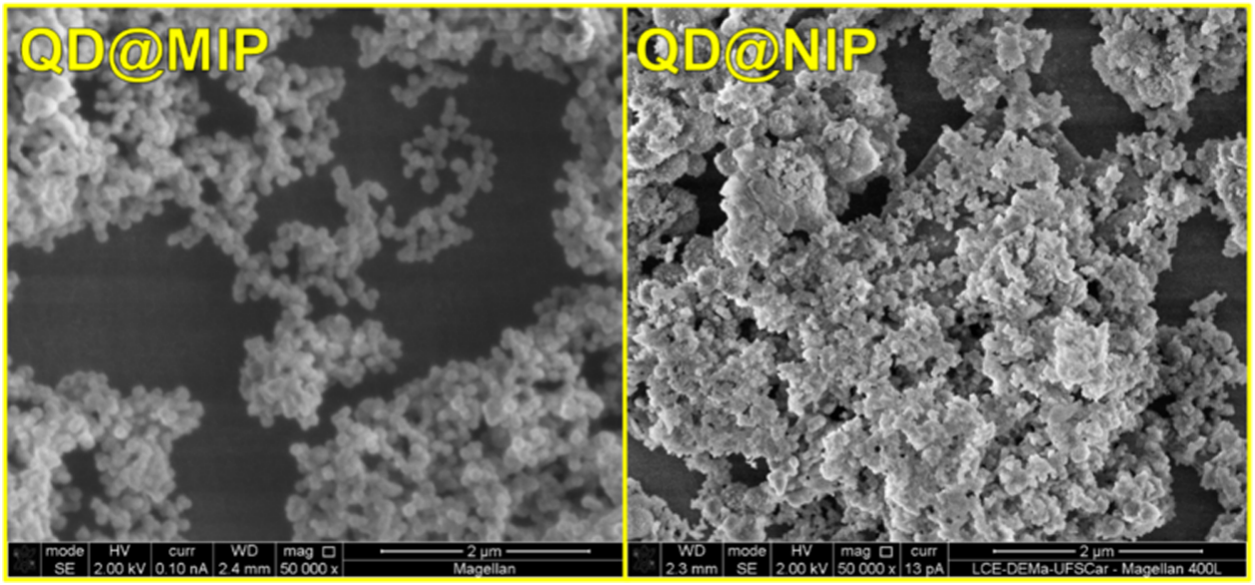
Scanning electron microscopic (SEM) images of the QD@MIP and QD@NIP.

Figure 1 illustrates the FEG-SEM morphological features of both QD@MIP and QD@NIP. Although both materials share similar structural characteristics, the molecularly imprinted polymer (MIP) exhibits greater homogeneity, characterized by numerous uniform spherical particles evenly dispersed throughout the sample. Additionally, the MIP displays a higher surface roughness compared to the nonimprinted polymer (NIP), which is attributed to the presence of selective recognition cavities within its matrix.

Confocal microscopy was employed for further characterization of QD@MIP. As shown in Figure 2, topographic analysis of the polymer surfaces revealed notable three-dimensional differences between the molecularly imprinted polymer (QD@MIP) and its nonimprinted control (QD@NIP). The QD@MIP surface displayed increased roughness compared to the control, aligning with the anticipated presence of selective cavities in the imprinted material. Average roughness values were measured at 0.9 ± 0.3 μm for the NIP and 3.4 ± 0.2 μm for the MIP, based on four measurements (*n* = 4).

**Figure 2:**
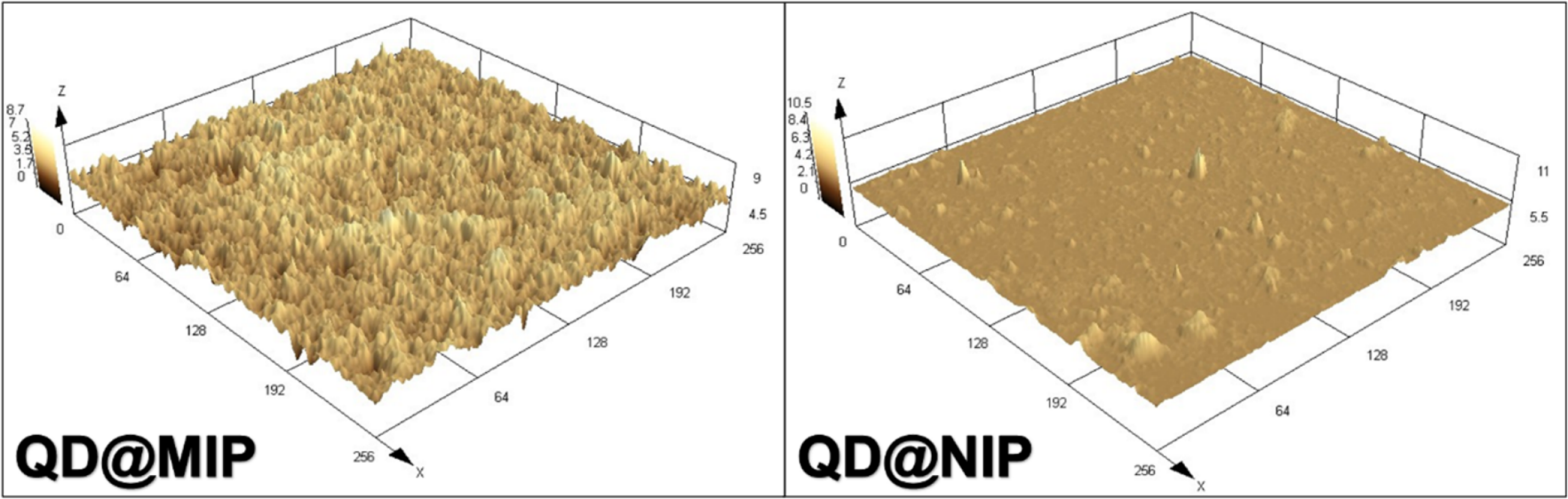
Confocal microscopy images illustrating the surface characteristics of QD@MIP and QD@NIP.

Fourier Transform Infrared Spectroscopy (FTIR) was employed to analyze the functional groups present in the polymers and quantum dots. Both QD@MIP and QD@NIP polymers were synthesized using identical reagents and similar procedures. Consequently, there is no difference in chemical composition between the MIP and the NIP, as both share the same formulation; the only distinction lies in the specific recognition cavities formed in the MIP during the polymerization process, which results in comparable FTIR spectra.

Figure 3 illustrates the spectra for QD@MIP, QD@NIP, and the structural monomer, tetraethyl orthosilicate (TEOS). A prominent band appears at 1045 cm^–1^ in both the imprinted and nonimprinted polymers, corresponding to the siloxane group (−Si–O–Si–) stretching modes, as documented in the literature.^28^ The TEOS spectrum also displays a similar band at 1043 cm^–1^, confirming that siloxane is present in the polymers, with TEOS as a primary structural component.

**Figure 3:**
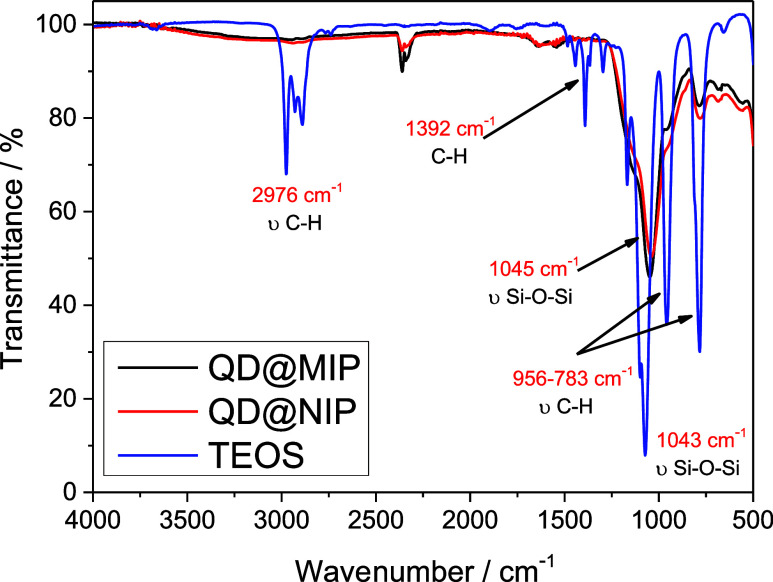
Infrared spectra of the QD@MIP, QD@NIP, and TEOS.

In the TEOS spectrum, the band at 2976 cm^–1^ is attributed to C–H stretching in ester groups, while the band at 1392 cm^–1^ corresponds to asymmetric C–H deformation. Additional bands at 956 and 783 cm^–1^ are also linked to C–H bonds within the molecule. However, in the MIP spectrum, these C–H-related bands are either significantly reduced or nearly absent, suggesting that C–H bonds are broken during polymerization.^29^

### 3.2 Optimization of the Sensor Response

The analyses of sulfathiazole in this work are based on the *fluorescence quenching* phenomenon of quantum dots (QDs), which occurs mainly through photoinduced electron transfer (PET) and Förster resonance energy transfer (FRET). In these interactions, sulfathiazole decreases the emission of QDs by accepting electrons or receiving energy, in addition to the possible formation of nonfluorescent static complexes. Furthermore, part of the light emitted by the QDs can be absorbed by sulfathiazole itself, resulting in a lower fluorescence signal detected by the fluorimeter. As the concentration of sulfathiazole increases, a progressive reduction in fluorescence is observed, enabling its sensitive detection and quantification.

To optimize the analytical conditions for the QD@MIP material, two key parameters were investigated: retention time—defined as the incubation period during which the imprinted polymer interacts with and adsorbs the analyte—and the pH of the solution, which influences the adsorption efficiency of QD@MIP.

To evaluate the optimal incubation time between QD@MIP and sulfathiazole (STZ), different durations (5, 10, 15, 30, 60, 90, and 120 min) were tested. The results, presented in Supporting Figure S1, show the variation in fluorescence intensity as a function of incubation time. Analysis of the data indicated that fluorescence intensity remained relatively stable across all time intervals, with no significant improvement observed beyond 5 min. Thus, an incubation time of 5 min was selected for subsequent experiments, as it provided a satisfactory analytical signal, reflecting effective analyte rebinding with adequate sensitivity in a short time frame.

The effect of pH on polymer-analyte interaction was assessed by preparing samples at pH values of 3, 5, 7, 9, and 11. Acidic and basic adjustments were made using 0.1 mol·L^–1^ HCl and 0.1 mol·L^–1^ NaOH, respectively. As shown in Figure 4, pH variation had no significant influence on fluorescence intensity, suggesting that the interaction between QD@MIP and STZ is not strongly pH-dependent. Therefore, pH adjustment of the sample solution is not required for effective use of QD@MIP.

**Figure 4:**
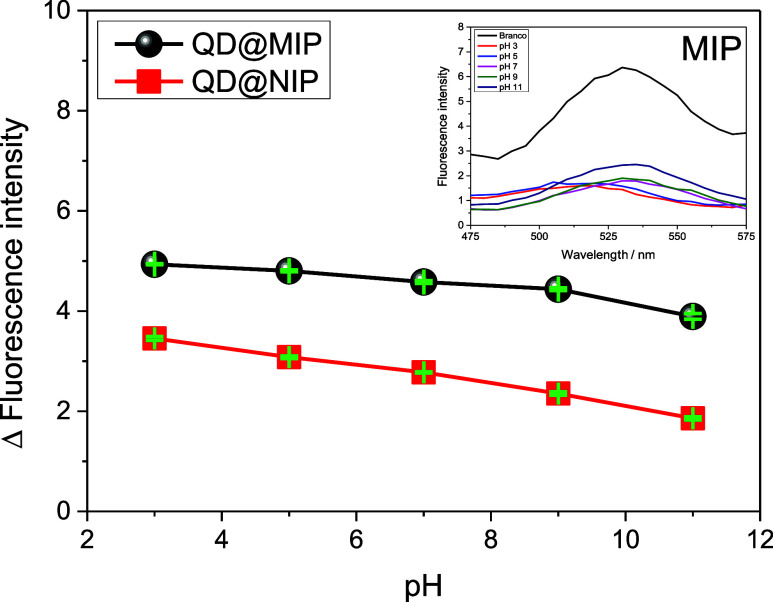
Graph of the fluorescence variation for the QD@MIP and QD@NIP about pH solution and an inserted QD@MIP fluorescence spectra. The measurements were made in triplicate using a 20 μg kg^–1^ STZ solution (the error bars are highlighted in green).

The pH difference is not significant for the purposes of this study, as the pH variation for the MIP was less than 1, according to the graph. Therefore, selecting the optimal pH for the MIP is more relevant than for the NIP. Maintaining a neutral pH is a more practical choice, as it requires fewer adjustments and is easier and faster to implement. Both analyses revealed that QD@MIP demonstrated a higher binding capacity than QD@NIP, attributed to the selective and sensitive cavities of the molecularly imprinted polymer coating on the quantum dot surface.

### 3.3 Response Profile of the QD@MIP

Following the optimization of rebinding time and solution pH, the next step involved constructing the analytical calibration curve. This curve was generated using varying concentrations of sulfathiazole (STZ) to determine the linear response range, assess the sensitivity, establish the linear regression equation, and calculate the limits of detection (LOD) and quantification (LOQ). Figure 5 presents the resulting analytical curves, illustrating the variation in fluorescence intensity as a function of STZ concentration for both QD@MIP and QD@NIP. These curves were derived from the corresponding fluorescence spectra shown in the associated graphs.

**Figure 5:**
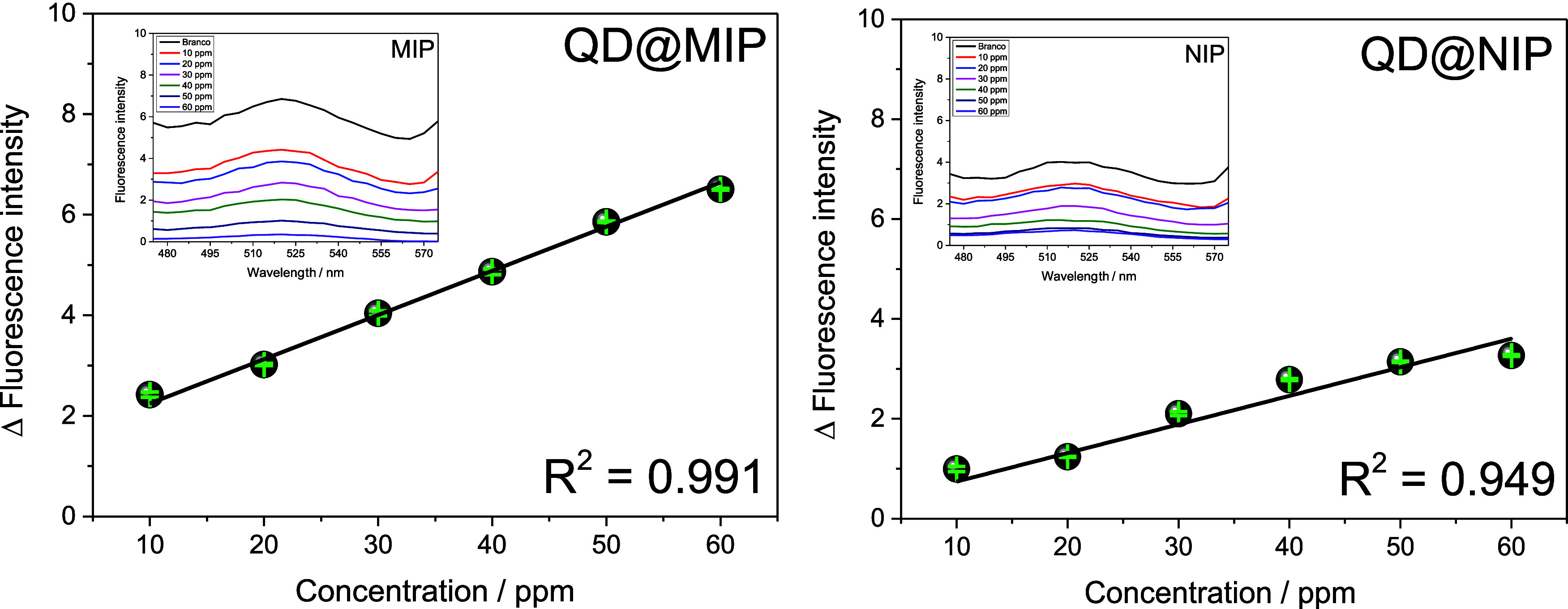
Analytical curves depicting the relationship between fluorescence intensity and concentration (ranging from 10 to 60 μg kg^–1^) for both QD@MIP and QD@NIP, with each measurement performed in triplicate (*n* = 3). The accompanying images display the fluorescence spectra of QD@MIP and QD@NIP obtained from varying concentrations of STZ dissolved in water, following the previously established experimental conditions. It is worth noting that the variation in fluorescence intensity was calculated as the difference between the fluorescence of the blank and that of the analyzed concentration.

Analysis of the fluorescence spectra for QD@MIP and QD@NIP, as presented in Figure 5, revealed that QD@MIP exhibited a more pronounced fluorescence variation. This indicates that the imprinted polymer was more effective in adsorbing the analyte compared to the nonimprinted polymer, which is a favorable and expected outcome. The observed decrease in fluorescence intensity with increasing STZ concentration confirms the quenching effect induced by the analyte. Fluorescence intensity measurements were collected at a fixed wavelength of 525 nm, corresponding to the maximum emission observed. Based on these values, analytical curves representing the fluorescence variation as a function of STZ concentration were constructed for both QD@MIP and QD@NIP.

The analytical curve for QD@MIP demonstrated good linearity within the concentration range of 10–60 μg·kg^–1^, surpassing the performance of its nonimprinted counterpart. The QD@MIP showed a correlation coefficient (*R* ^2^) of 0.991, while the QD@NIP curve yielded an *R* ^2^ of 0.949 for the same range. The linear equations corresponding to each calibration curve were obtained using OriginLab 9.0 software

$$y_{\mathrm{MIP}}=1.37\left(\pm 0.17\right)+0.087\left(\pm 0.004\right)x_{\mathrm{MIP}}$$

$$y_{\mathrm{NIP}}=0.17\left(\pm 0.22\right)+0.057\left(\pm 0.006\right)x_{\mathrm{NIP}}$$

From the linear regression equations, the slope (angular coefficient) and intercept (linear coefficient) were determined for both QD@MIP and QD@NIP. Additionally, based on the calculated sensitivity (*s*), the limits of detection (LOD) and quantification (LOQ) were estimated for each polymer. All corresponding values are summarized in Table 1.

**Table 1:** Parameter Data Obtained in the Analytical Curves of the MIP and NIP Polymers

parameter	QD@MIP	QD@NIP
response range (μg kg^–1^)	10.0–60.0	10.0–60.0
angular coefficients (sensitivity)	0.087	0.057
linear coefficients	1.37	0.17
limit of Detection (μg kg^–1^)	0.59	2.23
limit de Quantification (μg kg^–1^)	1.79	6.75

The limit of detection (LOD) and limit of quantification (LOQ) for QD@MIP were determined to be 0.59 and 1.79 μg·kg^–1^, respectively. These values demonstrate the high sensitivity of the sensor, enabling reliable detection of the analyte at low concentrations via fluorescence measurements. Furthermore, QD@MIP exhibited a significantly greater fluorescence variation than QD@NIP, consistent with the expected selective recognition behavior of the imprinted polymer.

### 3.4 Evaluation of Repeatability and Reproducibility

Repeatability studies refer to the consistency of results obtained from successive measurements using the same method. These measurements are performed under identical procedural conditions: by the same analyst, using the same equipment or instrument, and maintaining constant parameters such as temperature, pH, time, and concentration. All measurements and repetitions are conducted within a short time interval.^29,30^ In this study, repeatability was evaluated by performing ten consecutive measurements for each concentration using both QD@MIP and QD@NIP sensors, with identical samples and optimized conditions. The results, calculated using the same RSD equation, are presented in Table S1 and Figure S2 of the Supporting Information.

The findings in Table S1 indicate that QD@MIP exhibited a relative standard deviation of less than 6%, reflecting strong repeatability and outperforming QD@NIP in efficiency. The equations for the analytical curves below, obtained from Figure S2, along with their corresponding linearity coefficients (*R*), are as follows: 0.995 for QD@MIP and 0.973 for QD@NIP

$$y_{\mathrm{MIP}}=0.378\left(\pm 0.103\right)+0.071\left(\pm 0.002\right)x_{\mathrm{MIP}}$$

$$y_{\mathrm{NIP}}=0.901\left(\pm 0.125\right)+0.040\left(\pm 0.003\right)x_{\mathrm{NIP}}$$

Reproducibility studies were conducted by analyzing a single sample under various operating conditions (such as equipment, materials, locations, and analysts).^30^ In this study, reproducibility was evaluated by employing three different QD@MIP sensors and three different QD@NIP sensors on the same samples while maintaining identical optimized conditions. The results, calculated using the same RSD equation, are shown in Table S2 and Figure S3 of the Supporting Information.

The data in Table S2 reveal that QD@MIP achieved a relative standard deviation of less than 9%. While this is less favorable than the repeatability RSD, it still indicates a relatively acceptable result and demonstrates a good efficiency compared to the three QD@NIPs. The equations for the analytical curves obtained, along with their respective linearity coefficients (*R*) of 0.903 and 0.728, are provided below

$$y_{\mathrm{MIP}}=2.55\left(\pm 0.223\right)+0.037\left(\pm 0.005\right)x_{\mathrm{MIP}}$$

$$y_{\mathrm{NIP}}=2.13\left(\pm 0.183\right)+0.013\left(\pm 0.004\right)x_{\mathrm{NIP}}$$

### 3.5 Selectivity Analysis

To evaluate the effective formation of selective cavities in QD@MIP and confirm its molecular recognition capabilities, the sensor’s selectivity was assessed in the presence of other compounds, both structurally related and unrelated to sulfonamides. Four additional substances were tested: tetracycline (TTC), hydrochlorothiazide (HCT), chloramphenicol (CRP), and caffeine (CAF). These compounds were chosen due to their pharmaceutical relevance and their known quenching behavior within the same emission wavelength range as the quantum dots. The selectivity of the sensor was evaluated against potentially interfering compounds, as shown in Figure 6 below, another result is presented in Figure S4, in the Supporting Information section. It can be observed that the QD@MIP material exhibits a significantly higher response toward STZ compared to the other analytes and the control material (QD@NIP), indicating high selectivity.

**Figure 6:**
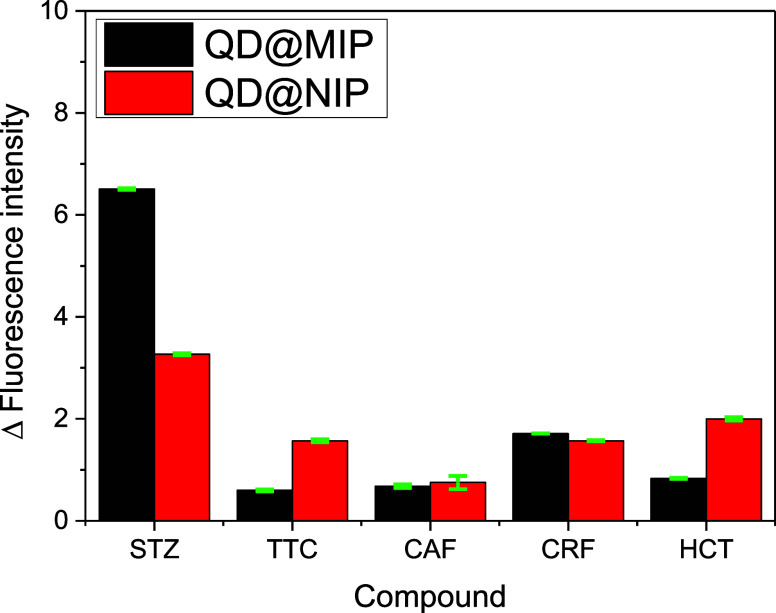
Comparative analysis was conducted using QD@MIP and QD@NIP for the determination of sulfathiazole and interferents. Measurements were taken separately at a concentration of 60 μg kg^–1^, in triplicate, at pH 7.0, with a 5 min incubation period.

To quantitatively assess the sensor’s selectivity, the molecular imprinting factor (α) and the selectivity factor (β) were calculated using the equations provided below, as documented in the literature for chemical sensors utilizing molecularly imprinted polymers^31^

$$\alpha =\frac{\Delta \mathrm{fluorescence}\;\left(\mathrm{QD}@\mathrm{MIP}\right)}{\Delta \mathrm{fluorescence}\;\left(\mathrm{QD}@\mathrm{NIP}\right)}$$

$$\beta =\frac{\alpha \left(\mathrm{STZ}\right)}{\alpha \left(\mathrm{Interferent}\right)}$$

The results of the selectivity analysis were both promising and satisfactory for the sulfathiazole QD@MIP. The proposed sensor exhibited enhanced recognition of the target sulfonamide even in the presence of potential interfering compounds. Notably, QD@NIP displayed a comparatively stronger response to the interferents than QD@MIP, reinforcing the specificity of the molecularly imprinted polymer. Despite this, both systems demonstrated markedly higher selectivity for sulfathiazole than for any of the interfering substances.

The molecular imprinting and selectivity parameters are summarized in Table 2. These results confirm that QD@MIP exhibits a strong affinity for sulfathiazole, attributed to the specific recognition sites created during the imprinting process.

**Table 2:**
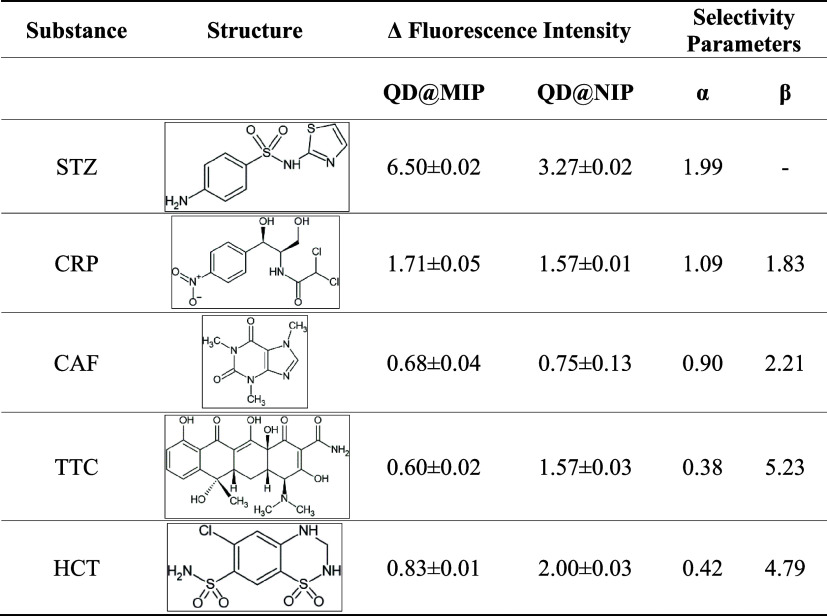
Values Derived from the Selectivity Parameters, Based on the Application of Sulfathiazole QD@MIP in Comparison to QD@NIP, Were Assessed in the Presence of Various Interfering Substances

The molecular imprinting factor (MIF) is defined as the ratio between the fluorescence variation observed for QD@MIP and that for QD@NIP. A higher MIF indicates a greater distinction in analyte recognition between the imprinted and nonimprinted polymers, reflecting the effectiveness of the molecular imprinting process.^31^ As shown in Table 2, the MIF for sulfathiazole was significantly greater than 1, confirming that QD@MIP adsorbed substantially more STZ than QD@NIP. This result is both expected and satisfactory, demonstrating the successful development of selective binding sites within the imprinted polymer.

For the interfering substances, the response observed with QD@NIP was equal to or greater than that of QD@MIP, resulting in MIF values close to or below 1. This indicates negligible selectivity between the polymers for these nontarget compounds, further confirming the specificity of QD@MIP for sulfathiazole. It is also worth noting that the adsorption of the interferents did not increase proportionally with concentration, suggesting a lack of specific interaction between these molecules and the recognition sites of the imprinted polymer.

The selectivity factor is defined as the ratio between the molecular imprinting factor (MIF) of the target analyte and that of a given interferent. A value greater than 1 indicates that the material exhibits higher selectivity for the analyte relative to the interferent. The greater the selectivity factor, the more selective the QD@MIP is toward the sulfonamide, reflecting superior molecular recognition performance and analytical reliability.^29^ As shown in the table, all selectivity factors of the interfering substances were high and greater than 1, indicating that there was selectivity of the QD@MIP for the detection of their sulfathiazole.

### 3.6 Application of the QD@MIP in Food Samples

To evaluate the interaction between the imprinted polymer (QD@MIP) and the target analyte (sulfathiazole, STZ) in real matrices, experiments were conducted using animal-derived food products. All analytical parameters were previously optimized, and the materials were fully characterized. Three food samples were selected for analysis—skimmed milk, egg (white and yolk homogenized), and honey—all obtained from local supermarkets in Araraquara, São Paulo, Brazil.

Each sample was fortified with sulfathiazole at three predefined concentrations: 10, 30, and 60 μg kg^–1^. The analyses were carried out using 100 μg of the polymer dispersed in 3.0 mL of water. The food samples underwent pretreatment procedures as described in Section X. The optimized conditions—5 min of interaction time and a solution pH close to 7—were applied to all experiments. The fluorescence responses obtained under these conditions are presented in Table 3.

**Table 3:** Results Obtained from Fluorescence Measurements Based on the Application of Sulfathiazole in Three Samples of Animal-Derived Foods

	fluorescence variation					
	milk		egg		honey	
concentration (μg kg^–1^)	MIP	NIP	MIP	NIP	MIP	NIP
10.0	2.22	0.23	2.33	0.23	2.26	0.59
30.0	3.65	1.95	3.97	0.86	3.99	1.88
60.0	5.87	2.68	5.41	1.66	6.41	2.60

To assess the performance of QD@MIPs in actual samples, the Recovery (%) was calculated, which is the percentage measure of the interaction between the analyte and printed polymer that is recovered after mixing in the matrix of a real sample. The Percent Recovery equation is given by the ratio between the variation in fluorescence in real samples and the variation in fluorescence in the analytical curve

$$recovery\;percentage=\frac{{\Delta fluo}_{sample}}{{\Delta fluo}_{analytical\;curve}}$$

It is worth remembering that the value of the fluorescence variation is given by the difference between the blank solution fluorescence and that measured at a given concentration. If the recovery is less than 100%, it means that the interaction capacity between the analyte and QD@MIP was lower in the sample matrix, if it is greater than 100%, it means that the interaction was greater. Table 4 below shows the results of the Percentage of Recovery values found for each sample.

**Table 4:** Results Obtained from Recovery Percentages in the Application of STZ in Three Samples of Animal-Derived Foods

	recovery (%)					
	milk		egg		honey	
concentration (μg kg^–1^)	MIP	NIP	MIP	NIP	MIP	NIP
10.0	91.6	23.0	96.2	23.2	93.3	58.9
30.0	90.4	93.0	98.3	40.8	98.8	89.5
60.0	90.2	82.0	83.2	50.8	98.5	79.7

Analyzing the percentage values calculated in the previous table, it was observed that all results were satisfactory for the polymer printed with all food samples, where the majority of recovery percentages were above 90%, except the egg sample with a percentage between 80 and 90%, this is due to its complexity to be treated due to the high amount of proteins present. It was also observed that these percentages reduce as the concentration of the analytes increases, this is due to the saturation of the QD@MIP at higher concentrations of antibiotic. Additionally, it was observed that QD@NIP presents nonspecific interactions, typical of its nonselective nature, showing recovery values in all cases lower than those of QD@MIP, considering all the complexity of the food samples and the importance of its pretreatment.

The results indicated that the proposed QD@MIP displayed outstanding selectivity, showing a high adsorption capacity for sulfathiazole. This remarkable selectivity is due to the incorporation of molecularly imprinted polymers as the sensing component in the optical sensor’s design. The utilization of this selective MIP sensor underscores the potential of this developed method for application in samples relevant to environmental and public health concerns.

## Conclusions

In this work was possible to develop an unprecedented QD@MIP optical sensor for the antibiotic sulfathiazole, obtaining satisfactory results, which have been validated against a QD@NIP. In the morphological characterization studies was possible, to verify the morphology of the QD@MIP and QD@NIP polymers, showing the homogeneity and robustness of the printed polymer with its selective cavities, as well as measuring its diameters. Confocal Microscopy calculated the roughness and porosity of printed materials. In the characterization of the QD@MIP by FTIR, the observed bands confirmed the presence of the polymer on the surface of the quantum dot. When analyzing the rebinding capacity of QD@MIP with sulfathiazole, the printed polymer showed great efficiency in determining the antibiotic, since MIP showed good rebinding to the selective cavities against other interfering substances. The sensor was optimized, and the detection and quantification limits were 0.59 and 1.79 μg kg^–1^ respectively. In the repeatability and reproducibility studies, all results presented an RSD below 9%, which is an excellent result of the sensor’s reliability in measurements and the reproducibility of polymerization in the construction of the QD@MIP. In the selectivity studies, it was possible to calculate the α and β parameters, obtaining values demonstrating that the constructed QD@MIP was highly selective for its analyte about the other interferents. Finally, when applying QD@MIP to real samples of food derived from animals, the results showed a good percentage of recovery, even under the effect of the sample matrix, indicating the material’s functionality, requiring only a presimple and fast sample processing.

## Supplementary Material



## Notes

Graph of the fluorescence variation for the QD@MIP and QD@NIP (Figure S1); repeatability values calculated for the QD@MIP and QD@NIP (Table S1); results obtained for repeatability of the QD@MIP and QD@NIP (Figure S2); reproducibility values calculated for the QD@MIP and QD@NIP (Table S2); results of reproducibility of the QD@MIP and QD@NIP (Figure S3); fluorescence variation curves and interferents for the QD@MIP sensor (Figure S4) (PDF)
