# Supplementary material for: Advanced Fluorometric Detection of Sulfathiazole Antibiotics in Food Samples with Molecularly Imprinted Polymer Coated CdTe Quantum Dots
Source: ACS Omega. 2025 Jul 23;10(30):33910–20. doi: 10.1021/acsomega.5c04765 (PMC12332599; doi:10.1021/acsomega.5c04765)

Supplementary Materials

Advanced Fluorometric Detection of Sulfathiazole Antibiotics in Food Samples with Molecularly Imprinted Polymer-Coated CdTe Quantum Dots

Bianca Mortari[a,b], Ademar Wong[a,b], Sabir Khan[c], Rosa Fireman Dutra[d] ,Maria Del Pilar Taboada Sotomayor[a,b*]**

[a]Department of Analytical Chemistry, Institute of Chemistry, São Paulo State University (UNESP), 14801-970 Araraquara, SP, Brazil

[b]National Institute for Alternative Technologies of Detection, Toxicological Evalucation and Removal of Micropollutants and Radioactives (INCT-DATREM), Araraquara, SP, Brazil

[c]Technological Development Center—CDTec, Postgraduate Program in Materials Science and Engineering, PPGCEM/UFPEL, Federal University of Pelotas, UFPel, CEP: 96010-610, Pelotas, RS, Brazil[d]Laboratory of Biomedical Engineering, Department of Biomedical Engineering, Federal University of Pernambuco, 50679-901 Recife, Brazil

Corresponding authors' address: **Corresponding author: mpilarts@hotmail.com*

***Corresponding author: sabir-chemist"yahoo.com*

Figure S1 - Graph of the fluorescence variation for the QD@MIP and QD@NIP about incubation time and an inserted QD@MIP fluorescence spectra. The measurements were made in triplicate using a 20 ppm STZ solution.

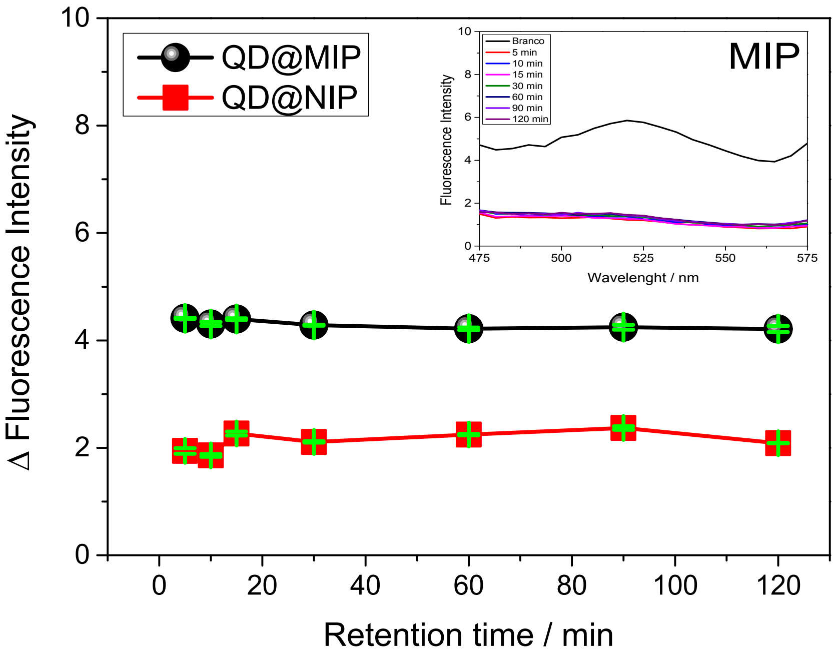

Table S1 - Repeatability values calculated for the QD@MIP and QD@NIP (n = 10).

	**QD@MIP**		**QD@NIP**	
Concentration (ppm)	Average (n = 10)	RSD (%)	Average (n = 10)	RSD (%)
10	1.14±0.07	5.93	1.22±0.11	9.13
20	1.82±0.05	3.01	1.93±0.13	6.80
30	2.47±0.08	3.30	2.12±0.11	5.12
40	3.10±0.06	1.87	2.40±0.09	3.92
50	4.19±0.09	2.17	2.84±0.07	2.63
60	4.66±0.04	0.87	3.36±0.09	2.84

Figure S2 - Results obtained from the analysis of repeatability of the QD@MIP and QD@NIP with the RSD (n = 10).

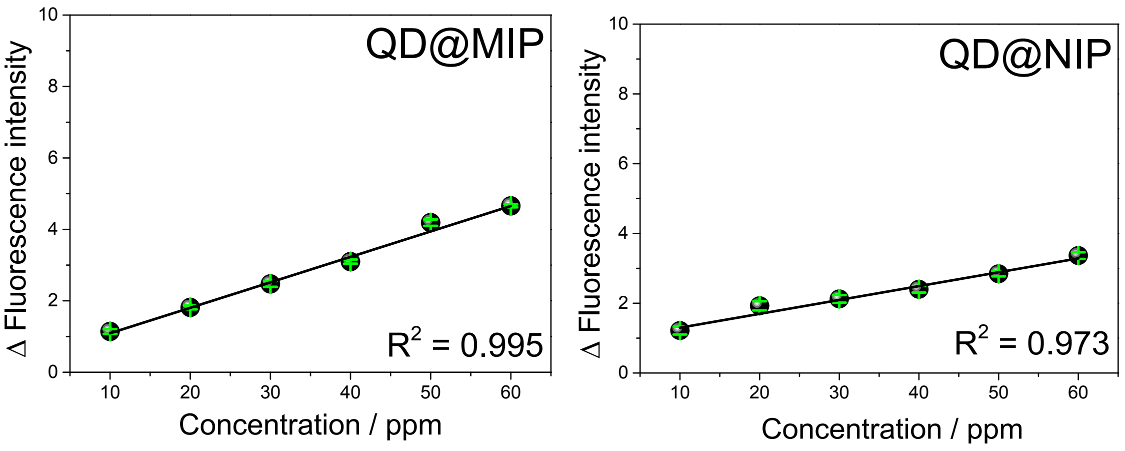

Table S2 - Reproducibility values calculated for the QD@MIP and QD@NIP (n = 3).

	**QD@MIP**		**QD@NIP**	
Concentration (ppm)	Average (n = 3)	RSD (%)	Average (n = 3)	RSD (%)
10	2.67±0.23	8.63	1.33±0.25	18.8
20	3.07±0.23	7.46	2.06±0.09	4.74
30	3.63±0.19	5.34	2.47±0.12	4.68
40	4.09±0.05	1.25	2.69±0.02	0.69
50	4.35±0.16	3.70	2.82±0.01	0.14
60	4.56±0.14	3.02	2.90±0.02	0.70

Figure S3 - Results obtained from the analysis of reproducibility of the QD@MIP and QD@NIP with the RSD (n = 3).

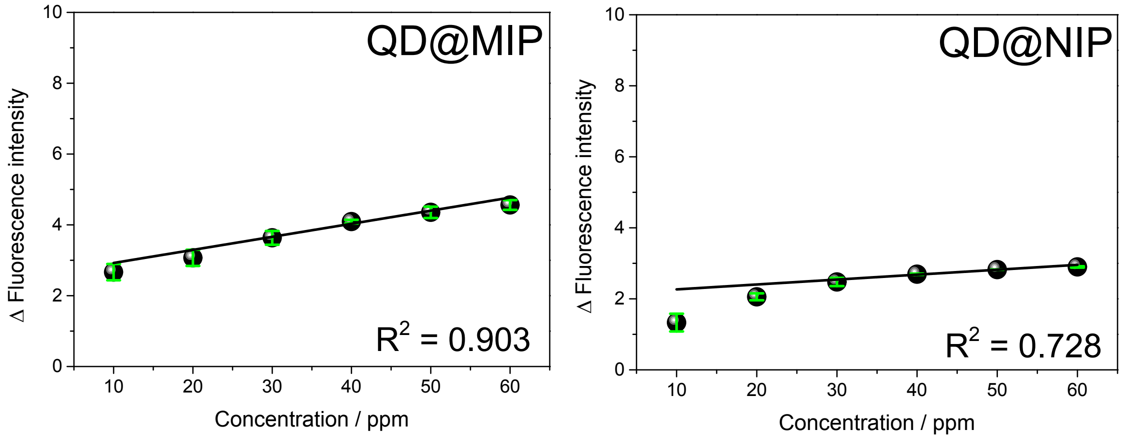

Figure S4 - Fluorescence variation curves as a function of concentration (10, 30, and 60 ppm in triplicate) of sulfathiazole and interferents for the QD@MIP sensor, measured separately.

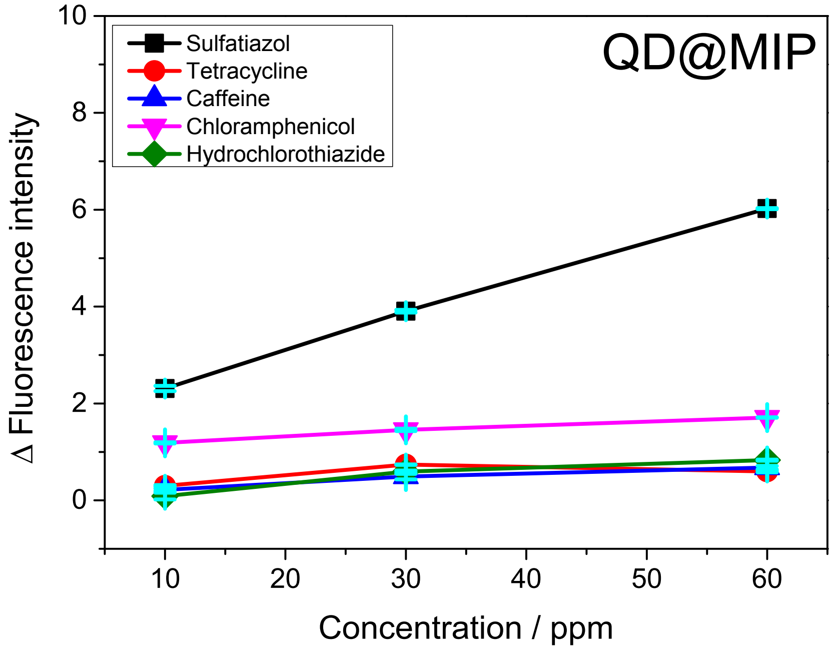

Supplement: Supplementary file 1 [file ao5c04765_si_001.pdf]
